# Recent advances in understanding the mechanisms of cerebellar granule cell development and function and their contribution to behavior

**DOI:** 10.12688/f1000research.15021.1

**Published:** 2018-07-26

**Authors:** Elizabeth P. Lackey, Detlef H. Heck, Roy V. Sillitoe

**Affiliations:** 1 Department of Pathology & Immunology, Baylor College of Medicine, Houston, TX, USA; 2 Department of Neuroscience, Baylor College of Medicine, Houston, TX, USA; 3 Jan and Dan Duncan Neurological Research Institute at Texas Children’s Hospital, 1250 Moursund Street, Suite 1325, Houston, TX, 77030, USA; 4 Department of Anatomy and Neurobiology, University of Tennessee Health Science Center, 855 Monroe Avenue, Memphis, TN, 38163, USA; 5 Program in Developmental Biology, Baylor College of Medicine, Houston, TX, USA

**Keywords:** Cerebellum, granule cell, Purkinje cell, development, in vivo electrophysiology, behavior

## Abstract

The cerebellum is the focus of an emergent series of debates because its circuitry is now thought to encode an unexpected level of functional diversity. The flexibility that is built into the cerebellar circuit allows it to participate not only in motor behaviors involving coordination, learning, and balance but also in non-motor behaviors such as cognition, emotion, and spatial navigation. In accordance with the cerebellum’s diverse functional roles, when these circuits are altered because of disease or injury, the behavioral outcomes range from neurological conditions such as ataxia, dystonia, and tremor to neuropsychiatric conditions, including autism spectrum disorders, schizophrenia, and attention-deficit/hyperactivity disorder. Two major questions arise: what types of cells mediate these normal and abnormal processes, and how might they accomplish these seemingly disparate functions? The tiny but numerous cerebellar granule cells may hold answers to these questions. Here, we discuss recent advances in understanding how the granule cell lineage arises in the embryo and how a stem cell niche that replenishes granule cells influences wiring when the postnatal cerebellum is injured. We discuss how precisely coordinated developmental programs, gene expression patterns, and epigenetic mechanisms determine the formation of synapses that integrate multi-modal inputs onto single granule cells. These data lead us to consider how granule cell synaptic heterogeneity promotes sensorimotor and non-sensorimotor signals in behaving animals. We discuss evidence that granule cells use ultrafast neurotransmission that can operate at kilohertz frequencies. Together, these data inspire an emerging view for how granule cells contribute to the shaping of complex animal behaviors.

## Introduction

**Figure 1.**
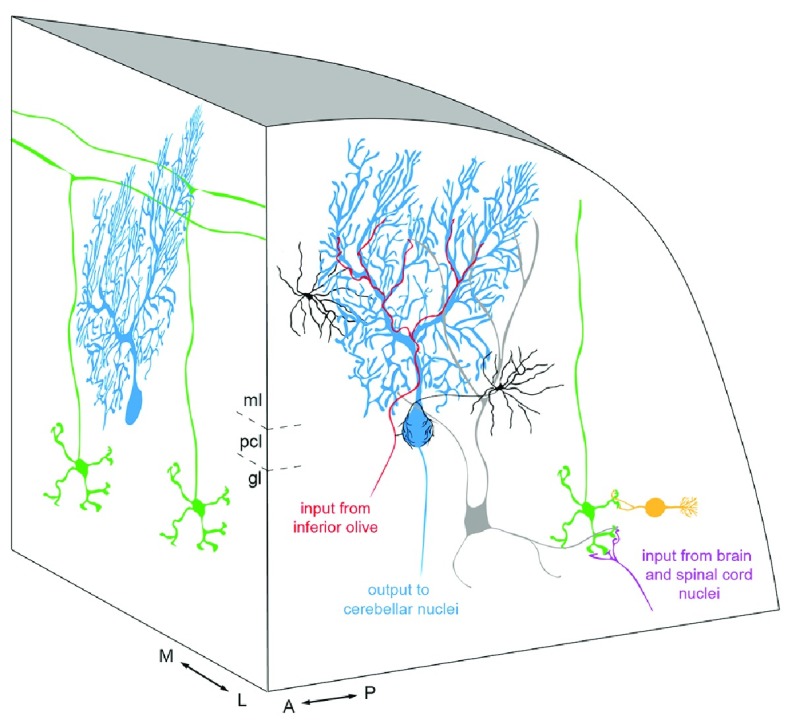
Architecture of the cerebellar cortical circuit. A three-dimensional schematic of the cerebellar cortex illustrates the repeating basic circuit of Purkinje cells (blue), granule cells (green), climbing fiber afferents (red), mossy fiber afferents (magenta), stellate and basket cell interneurons (black), Golgi cell interneurons (gray), and unipolar brush cell interneurons (orange). Granule cell axons ascend to bifurcate into parallel fibers along the mediolateral axis that contact the dendrites of Purkinje cells. Purkinje cell axons deliver the output of the circuit to the cerebellar nuclei, which are the final output of the cerebellum to the rest of the nervous system. However, a small number of Purkinje cells project directly to the vestibular nuclei. For simplicity, we have not shown Lugaro cells or candelabrum cells. A, anterior; gl, granular layer; M, medial; ml, molecular layer; L, lateral; P, posterior; pcl, Purkinje cell layer.

The cerebellum is well known for its roles in motor functions, including coordination, posture, balance, and learning ^1– 4^. However, recent work shows strong support for cerebellar contributions to non-motor functions such as cognition, emotion, and language ^5– 7^. Studies that have examined the normal operation of the cerebellum are in accordance with its involvement in a variety of diseases. Structural and functional cerebellar abnormalities are associated with a number of conditions such as ataxia, tremor, dystonia, autism spectrum disorder, schizophrenia, and attention-deficit/hyperactivity disorder (for a recent review, see 8). While different genetic and molecular mechanisms distinguish the underlying causes of these disease states, in many instances cerebellar information processing is affected. More specifically, problems with information processing within the cerebellar cortex are a common theme across a number of neurological and neuropsychiatric conditions. It is therefore interesting to ask, is there a common denominator at the cellular and circuit levels, and how does it work in the healthy brain? Here, we review recent studies demonstrating how the cerebellar input layer, which contains the most numerous neuronal type in the brain, cerebellar granule cells ( Figure 1), could contribute to such processes in health and disease. Specifically, we discuss work from multiple disciplines focused on the development, connectivity, electrophysiology, behavior, and disease contribution of the granule cells. These new data on granule cells add to the rapidly expanding view of how the cerebellum functions during motor and non-motor behaviors.

## A primer on granule cell microcircuitry

**Figure 2.**
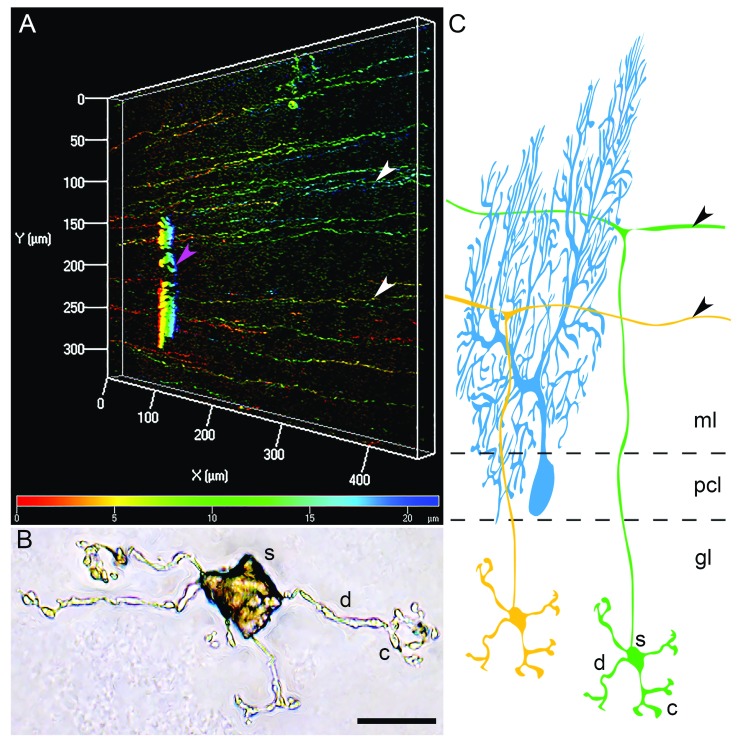
Morphology of the granule cell axons and dendritic claws. ( **A**) Genetically labeled parallel fibers (white arrowheads) traversing the molecular layer along the mediolateral axis. A portion of a genetically labeled Purkinje cell dendritic arbor is also visible (magenta arrowhead). In this three-dimensional reconstruction, the pseudocoloring encodes the depth along the z-axis of the tissue section; the red pseudocolor indicates structures that are closest to the surface, and the blue pseudocolor indicates deeper structures. ( **B**) A single granule cell labeled using the Golgi-Cox method. The dendrites and claws are revealed in exquisite detail. Scale bar = 10 μm. ( **C**) A schematic depicting the structures shown in ( **A**) and ( **B**). Black arrowheads point to parallel fibers. c, claw; d, dendrite; gl, granular layer; ml, molecular layer; pcl, Purkinje cell layer; s, soma.

One often-cited claim to fame of the cerebellum is that it contains more than half of all the neurons in the mammalian nervous system. It owes this designation entirely to the miniscule granule cells, which form the innermost cell layer of the cerebellar cortex, appropriately called the granular layer ( Figure 1). To fully appreciate the recent advances in understanding granule cell function, it is worth briefly revisiting their microcircuitry. Located above the granular layer are two additional layers: the Purkinje cell layer and the molecular layer. These three layers contain the neurons that form the canonical microcircuit of the cerebellar cortex. The Purkinje cell layer contains the cell bodies of the large Purkinje cells, which are the main computational cell type of the cerebellum and the sole output of the cerebellar cortex. The Purkinje cell layer also contains the cell bodies of candelabrum cells and a specialized type of astrocyte called the Bergmann glia. All three cell types extend their processes into the molecular layer, the most superficial layer. This layer also contains the inhibitory stellate and basket cell interneurons. Granule cells project an axon that ascends past the Purkinje cell somata into the molecular layer, where it bifurcates to form the parallel fibers, which form excitatory contacts on the Purkinje cell dendrites. Parallel fibers also contact the molecular layer interneurons. The granule cell dendrites, typically three to five per granule cell, extend locally around the granule cell somata and are recognized by their characteristic claw-like appearance ( Figure 2). The granule cell dendrites receive excitatory input from mossy fiber afferents as well as inhibitory axon terminals from Golgi cells. Golgi cells, with their cell bodies located within the granular layer and their dendrites reaching into the molecular layer, are ideally positioned for interaction with parallel fibers originating from the same granule cells that they inhibit ( Figure 1). The granule cell dendrite, Golgi cell axon terminal, and mossy fiber terminal form the core of a structure called a glomerulus. The granular layer also contains inhibitory Lugaro cells, and the posterior-most lobules have a dense accumulation of excitatory unipolar brush cells ( Figure 1). Unipolar brush cells project directly to granule cells and serve to amplify vestibular inputs to the cerebellum. An added level of specificity is that granule cells in vestibular and eye movement regions also receive direct inhibitory input from Purkinje cell axon collaterals ^9^. Interestingly, granule cells and unipolar brush cells are derived from a common developmental lineage ^10– 12^. Also arising from this lineage are the excitatory projection neurons of the cerebellar nuclei, which are the target cells for Purkinje cell axons. Cerebellar nuclear neurons produce the main output of the cerebellum to the rest of the nervous system. There are three bilateral pairs of cerebellar nuclei: the fastigial nucleus is most medial, the interposed nucleus is in the middle, and the dentate nucleus is most lateral. Each nucleus contains GABAergic, glycinergic, and glutamatergic neurons. The source of the glutamatergic neuron lineage is a specialized germinal structure called the rhombic lip. We start by considering the rhombic lip and the embryonic origins of granule cells as an avenue to discussing cerebellar function and behavior.

## Granule cells are derived from a complex genetic program that initiates during embryogenesis

**Figure 3.**
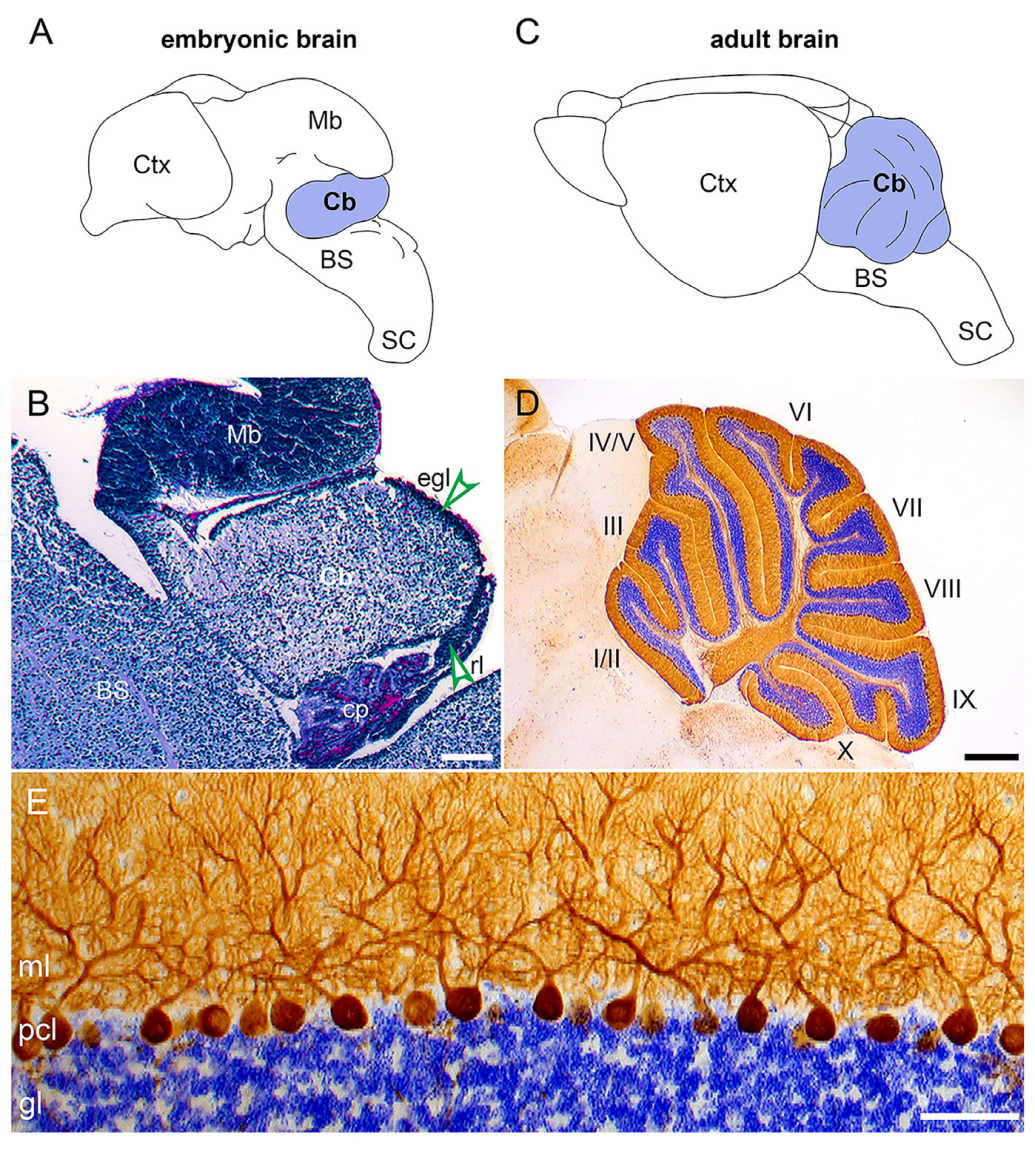
Development of the cerebellar cortical layers. ( **A**) A schematic depicting the embryonic brain with the cerebellum highlighted in color. ( **B**) A sagittal section from an embryonic day 16 brain with the cell bodies of neurons labeled using a Nissl stain (violet). The densely labeled external granular layer (green arrowhead, egl) and rhombic lip (green arrowhead, rl) are visible. ( **C**) A schematic depicting the adult brain with the cerebellum highlighted in color. ( **D**) A sagittal section from an adult brain with the cell bodies of neurons labeled using a Nissl stain (violet) and Purkinje cells labeled using calbindin immunohistochemistry (brown). The densely labeled granule cells are visible in the innermost layer of the cerebellar cortex, and the Purkinje cell somas and dendrites are visible in the outer layers of the cerebellar cortex. Roman numerals identify the 10 lobules. ( **E**) A magnified view of ( **D**). Scale bars = ( **B**) 100 μm, ( **D**) 500 μm, and ( **E**) 50 μm. BS, brainstem; Cb, cerebellum; cp, choroid plexus; Ctx, cerebral cortex; gl, granular layer; Mb, midbrain; ml, molecular layer; pcl, Purkinje cell layer; SC, spinal cord.

In the late 1890s through to the early 1900s, His ^13^ and then Essick ^14^ identified a region with peculiar developmental properties in the dorsal hindbrain of human embryos. Based on basic morphology and histology, they found that although this region was continuous with the rest of the hindbrain, it had a distinct anatomy and contained populations of cells that were mitotically active in the late stages of embryogenesis. They also noted that this region produced cells that migrated in streams away from the source. His referred to this dorsal germinal zone, which sits adjacent to the fourth ventricle, as the *rautenlippe* ^13^, or rhombic lip ( Figure 3B), because of its resemblance to lips on an open mouth. Note that the base of the fourth ventricle contains the other cerebellar germinal zone called the ventricular zone. More recent work using the transcription factor-encoding gene *Atoh1* ( *Math1*) demonstrated not only molecular specificity of the rhombic lip ^15^ but also that this gene is required for the production of granule cells ^16^. *Atoh1* is not the only gene whose expression is specific for the rhombic lip. Other genes such as *Lmx1a*, *Pax6*, *Tbr2*, *Cux2*, and *Wls* compartmentalize the rhombic lip into distinct molecular domains ^17– 20^. The elegant use of quail-chick chimeras allowed a careful analysis of how granule cell precursors are produced and migrate away from the rhombic lip to populate the external granular layer ( Figure 3B) ^21^. The external granular layer is a secondary germinal zone that produces the millions of granule cell progenitors that will later differentiate and then migrate into the core of the cerebellum using Bergmann glia astrocytes as a guide ^22^. Sonic hedgehog (Shh) ^23^, through Gli2 signaling ^24^, is required for granule cell proliferation, and recent work shows that the transcription factor Meis1 controls granule cell precursor differentiation through a Pax6–Bmp pathway that functions to degrade Atoh1 ^25^. One of the major breakthroughs in the field was the demonstration that the rhombic lip gives rise to more than just the granule cells. A combination of knock-in and knock-out mice as well as genetic inducible lineage-tracing techniques was used to show that the rhombic lip also produces the projection neurons of the cerebellar nuclei and the unipolar brush cells ^10– 12, 19^. These data indicated that the rhombic lip produces all of the glutamatergic neuronal classes of the cerebellum—a finding that was supported by showing that the ventricular zone produces all the GABAergic neurons of the cerebellum ^26^. The specification of neuronal fates and the identities of the inhibitory versus excitatory neuronal classes are maintained by *Ptf1a* function in the ventricular zone and *Atoh1* in the rhombic lip ^27^. Indeed, genetic removal of *Ptf1a* is enough to transform ventricular zone progenitors into a granule cell-like phenotype ^28^. Taken together, the genetic cascades that orchestrate the embryonic stages of granule cell development set in place a morphogenetic program that allows the cerebellum to grow in size, organize its circuitry, and attain its characteristic folded morphology ( Figure 3).

## Granule cells are critical for postnatal morphogenesis

The folded external gross morphology of the cerebellar cortex into lobules is a conserved feature that makes this structure easily recognizable in warm-blooded vertebrates ^29^. There are 10 major lobules ( Figure 3D), although depending on the species each one can be further divided into a given number of sublobules. If granule cells are not produced correctly, the growth and patterning of the cerebellum into different lobules are severely disrupted ^23, 30, 31^. In theory, such disruptions could be rescued by stem cells, which are found in the embryonic, postnatal, and adult cerebellum ^32, 33^. But a recent study uncovered that a *Nestin*-positive progenitor population in the early postnatal cerebellum is capable of replenishing the granule cell precursor population after acute depletion of the postnatal external granular layer ^34^. However, the consequences of over-producing cells that come from the rhombic lip lineage can be catastrophic. The result could be medulloblastoma, the most common pediatric brain cancer. Granule cell precursors are the main cell of origin of the Shh subgroup of medulloblastoma ^35^. Interestingly, tumors develop preferentially from the hemispheres, and the difference in sensitivity could be linked to intrinsic genetic differences in the granule cell precursors that reside in the hemispheres compared with the vermis ^36^. Regardless of their mediolateral origin in the cerebellum, Atoh1 regulates Shh signaling in medulloblastoma, as demonstrated by the suppression of tumor formation when the *Atoh1* gene is deleted ^37^. Conversely, loss of the 30-zinc finger transcription factor Zfp423 causes ciliopathy-related phenotypes such as vermis hypoplasia in Joubert syndrome ^38^. Zfp423 is expressed in the granule cell precursors and its deletion eliminates Shh responsiveness, resulting in a decline in granule cell proliferation. The molecular signaling pathways that are initiated when Shh function is depleted or in Shh-mediated medulloblastoma are complex ^39^, but, from a neural function perspective, it is the wiring in the cerebellar cortex that is of major relevance to the operation of circuits during behavior. The rhombic lip generates temporally controlled waves of granule cell precursors that eventually settle into distinct anterior-posterior domains ^11^. This division in spatial settling pattern is consistent with the lineage boundaries revealed by using embryonic stem cell chimeras, gene expression, and the phenotypes of mutant mice ^40^. Moreover, this mode of spatial patterning is conserved, as a similar partitioning was recently reported in zebrafish ^41^. It could be that defects in these early compartmental events are responsible for the region-specific abnormalities in cerebellar foliation and layering in Dandy–Walker malformation ^42^. This is not the only level of compartmental organization in granule cells. They also fall into a pattern of mediolateral clusters that are thought to organize according to Purkinje cell stripes ^43^. Purkinje cells are born into an array of parasagittal stripes that shape the pattern of all other cerebellar neurons, including the granule cells and their mossy fiber inputs ^44^. Accordingly, altering Purkinje cell patterning leads to abnormal topography of mossy fiber afferents ^45, 46^. What is intriguing is that during embryonic and early postnatal development, mossy fibers make direct functional contacts onto Purkinje cells ^47– 49^. These transient contacts are patterned according to Purkinje cell stripes ^50^. As the granule cells differentiate, they migrate radially from the external granular layer past the Purkinje cells to the internal granular layer. In the internal granular layer, they settle into lobules on the basis of clonal history ^51^, although in each lobule clonally related granule cells are randomly positioned in the granular layer ^52, 53^. Along the way to the internal granular layer, the granule cells connect with mossy fibers, which switch synaptic partners from Purkinje cells. The mechanism for how this switch occurs is not known, although mossy fiber terminals are likely remodeled in the process and it therefore could involve axon guidance cues, secreted factors, and trophic support ^47, 54, 55^. Both these morphogenetically active mossy fibers and the Purkinje cells could control the patterning of granule cells ^56^. At the same time, intrinsic genetic and epigenetic mechanisms regulate the formation of granule cell dendrites in order for them to accommodate the incoming mossy fiber terminals ^57^. Interestingly, in addition to supporting the formation of mossy fiber synapses, granule cells are required for pruning climbing fiber to Purkinje cell synapses during postnatal circuit refinement ^58^. Therefore, the granule cells are central to a number of processes that are required for establishing the structure of the cerebellum and at a finer level for ensuring that its cellular components are correctly wired into circuits. Although we will not cover the details here, please refer to the many excellent works of Mary Beth Hatten, Alain Chedotal, Azad Bonni, Dan Goldowitz, Carol Mason, Pasko Rakic, Rosalind Segal, and others for how the cellular and molecular mechanisms of granule cell migration and differentiation have been resolved by using a number of elegant *in vitro* and *in vivo* preparations.

## The structural and functional synaptic architecture of granule cell inputs

The round cell bodies of granule cells measure only 5 to 10 µm across (labeled as “s” in Figure 2B). They project an ascending axon that bifurcates into parallel fibers in the molecular layer ( Figure 2B, C). The parallel fibers extend an average of 6 mm ^59, 60^. Each granule cell has on average four short (12 to 20 μm) dendritic processes (labeled as “d” in Figure 2B) that end in a claw-like appendage (labeled “c” in Figure 2B). The claw-like ending on each dendrite receives one synaptic input, typically a rosette formed by a mossy fiber terminal. Unipolar brush cell axons also terminate in mossy fiber-like rosettes that make synaptic contacts directly onto the dendritic claws of multiple granule cells ^61^. A granule cell would be predicted to receive this sparse synaptic input from within its rather limited radius of only about 20 μm ( Figure 2B). It is the convergence of mossy fiber inputs onto a single granule cell that is perhaps more reflective of their processing power. A combination of genetic marking and viral-mediated tracing in mouse showed anatomical evidence for convergent multi-modal mossy fiber input onto single granule cells ^62^. Based on the tracing of cuneocerebellar and pontocerebellar terminals, the authors concluded that individual granule cells simultaneously process sensory and motor signals. Building on that study, Häusser *et al*. demonstrated that functional convergence of multi-modal sensory inputs onto individual granule cells serves to enhance action potential output ^63^. An attractive problem to resolve would be whether the different combinations of convergence produce distinct granule cell output frequencies that cause the release of unique subpools of vesicles ^64^. These findings also raise questions about whether convergence influences the ultrafast kilohertz neurotransmission at mossy fiber-to-granule cell synapses ^65^ and how convergence impacts the subsequent high-frequency burst firing of the granule cells ^66, 67^, particularly during behaviors that require dynamic modulation ^68^. Using a combination of slice and *in vivo* preparations, Chabrol, DiGregorio, and colleagues found that signatures in synaptic strength and short-term plasticity represent different modalities of mossy fiber-to-granule cell inputs and that specific multi-modal convergences result in distinct output firing at the level of individual granule cells ^69^. It is also interesting to speculate how this level of synaptic specificity relates to a broader systems organization. For example, in the anterior vermis, the terminal field topography of mossy fibers from the dorsal spinocerebellar tract (hindlimb fine touch and proprioception) interdigitates with mossy fibers from the external cuneate nucleus (forelimb/shoulder proprioception and fine touch) ^70, 71^. But there are also domains where the terminal fields overlap. Here, the mossy fiber-to-granule cell pathway could favor combinatorial processing and pattern discrimination, as suggested by Albus ^72^, Ito ^73^, and Marr ^74^. However, because mossy fibers terminate in patterns that respect the Purkinje cell map, their organization could promote communication between cortical modules ^75^, since the parallel fibers project several millimeters and therefore cross multiple modules ^76^. And because the Purkinje cells in the different modules express different molecular markers ^77^, have specific neuronal firing properties ^78, 79^, and express distinct modes of synaptic plasticity ^80^, it is possible that parallel fibers multiplex modules to help execute complex behaviors. In such a model, synchronous neural activity within modules ^81, 82^ would facilitate robust communication between the cerebellar cortex and the cerebellar nuclei ^83, 84^. Activity-dependent flavoprotein imaging experiments indicate that granule cell function indeed operates within a modular framework ^85^, although it is not clear how parallel fiber-induced patterned activity drives behavior. However, there is some evidence that the interaction between patches of granule cells and Purkinje cells is shaped by the molecular layer interneurons and that the strength of this inter-layer communication is dependent on relative position to Purkinje cells in the sagittal and mediolateral axes ^86, 87^.

## Granule cells encode sensorimotor and non-sensorimotor information

Early speculations about the function of granule cells had focused on their sparse synaptic inputs and their enormous number. This idea resulted in the influential theory that the role of the granule cell input layer was to recode the mossy fiber input in such a way that each granule cell would represent a unique combination of mossy fiber activity and fire only sparsely to signal to the output neurons, the Purkinje cells, in the presence of a specific input pattern ^72, 74^. This idea could not be tested experimentally until very recently, when the use of genetically expressed calcium indicators combined with large-field two-photon imaging finally allowed the monitoring of activity in large populations of granule cells simultaneously in awake behaving animals. Results from three different labs and two different species (zebrafish and mice) provided groundbreaking insights into granule cell activity that are quite different from the expectation based on the classic theory ^88, 89^ though still with some evidence preserving the idea of sparse coding ^90^. Granule cells may not show the sparse activity patterns predicted by classic theory. In general, the imaging results showed that large populations of granule cells were active at any given time in response to a sensory stimulus or during performance of a motor task, such as fluid licking and lever pressing ^88– 90^. Knogler *et al*. took advantage of the transparency of the larval zebrafish preparation to image calcium activity in granule cells ^89^. Granule cell responses to visual stimulation and a mild electric shock and during swimming movement consisted of highly correlated population activity in dense rather than distributed groups of granule cells for each condition ^89^. However, there are features of the model system that should be considered. Major milestones of the anatomical and functional maturity of cerebellar circuits, as well as the ability to perform motor behaviors, are present in larval zebrafish ^89, 91^; however, the larval brain does not execute adult functions, and there are likely many features of granule cell function that have yet to mature. Giovannucci *et al*. characterized granule cell activity in awake mice while they learned the cerebellum-dependent conditioned eye-blink task ^88^. Their results were consistent with the findings by Knogler *et al*. ^89^ in that they also observed dense populations of granule cells being activated during movement and sensory stimulation. However, it should be noted that Giovannucci *et al*. ^88^ performed their recordings in awake mice. As a consequence, body movements which could occur during the eye-blink response would also contribute to the overall pattern of granule cell activity. This makes it difficult to draw a final conclusion about the sparseness of granule cell coding related to the eye-blink response alone. However, Giovannucci *et al*. found that some granule cells began representing the learned pre-emptive eyelid closure and that the number of activated granule cells increased as training continued. Classic theories limited cerebellar motor learning to the output stage and focused on plasticity at the parallel fiber-to-Purkinje cell synapse ^72, 74^. These new findings show that understanding cerebellar learning also needs to take granule cells into account, although we still do not know whether the observed learning-related changes result from intra-cerebellar plasticity or changes in mossy fiber activity or contributions from both. Interestingly, recent work by Carey *et al*. suggests that the activation of mossy fiber-to-granule cell synapses is crucial for changes in behavioral state to modulate cerebellar learning ^92^.

The experiments by Wagner *et al*., which were conducted in awake behaving mice, added the exciting new insights that populations of granule cells potentially encode different aspects of reward expectation and that the largest number of granule cells signal the absence of an expected reward ^90^. However, although the study clearly established a reward-related aspect of granule cell activity, the authors could not definitively disentangle licking-related activities and non-motor signals. Moreover, although one could argue that the selectivity of granule cell responses deviates from a sparsity code, the authors did report that only about 20% of all imaged granule cells were active during the lever push and that about 25% in total were active during the reward phases of the task ^90^. As with all behaviors, the anatomical substrate of the neural networks should be able to support the functions. A recent study by Gilmer and Person ^93^ used a detailed anatomical model of the cerebellar granular layer and of mossy fiber projection patterns to show that morphological constraints, details of which were not yet known at the time when classic theories were developed, predict a dense and clustered population activity of granule cells in response to mossy fiber inputs in patterns that are consistent with the recent experimental findings discussed above ^88– 90^. Rather than hastily abandoning sparse coding theories, the new findings perhaps indicate that we should continue to test the idea of granule cells using a sparse code to provide unique information about specific combinations of mossy fiber input patterns. In summary, the use of imaging techniques that allow observations of large populations of granule cells in behaving animals has revealed that granule cells are active in large, redundant groups and that their activity patterns change in time and space with learning. But the cellular mechanisms guiding these responses are still unclear. An important consideration is how we determine whether multiple granule cells are simultaneously active, which depends critically on the choice of the time interval that defines “simultaneous”. For example, if the temporal resolution for measuring granule cell population activity was higher than that of current methods such as calcium imaging, what appears to be synchronous population coding could turn out to be precisely timed spike activity of a sparse coding scheme.

Reward expectation-related activity is a new and intriguing aspect of granule cell function that expands the spectrum of cerebellar cognitive functions. It is interesting that these data may even shed some light on the long-suspected role of the cerebellum in addiction ^94^. However, it will be difficult to fully resolve the role of granule cells (or, for that matter, any component of cerebellar circuitry) in non-motor behavior ^95^. Two hurdles in solving this problem are that there does not seem to be a clear distinction between the cerebellar circuits that control motor versus non-motor behavior—it may be that the circuitry has a built-in flexibility—and that many of the non-motor paradigms that are used in rodents, typically the animal model of choice for studying behavioral tasks because of the available genetic tools for manipulation, often have a motor component, such as in the study by Wagner *et al*. Therefore, it is perhaps wise to deeply consider human studies to address the complex behavioral mysteries of the brain despite the limitations on experimental manipulations. Functional whole-brain imaging in humans indicates BOLD (blood oxygen level-dependent) responses in the cerebellar cortex during tasks such as perception, language, and emotion ^96^. These responses are topographically organized, with activity related to sensorimotor tasks localized to the anterior cerebellar cortex and activity related to cognitive and limbic tasks localized to the posterior cerebellar cortex ^5, 7, 97^. Furthermore, language-related activity is lateralized in the posterior cerebellar cortex ^7^. Damage to the human cerebellum can cause not only distinct motor symptoms such as ataxia, dystonia, tremor, and dysmetria but also numerous cognitive deficits depending on the location of the lesion ^6, 98^. The underlying neuronal mechanisms are still unknown, and it would be interesting to determine how granule cells in different regions are affected in each case. Alluding to the assumption that the cerebellum performs similar neuronal computations for motor and non-motor processes, Schmahmann has proposed the “dysmetria of thought” hypothesis ^99, 100^. This theory predicts that if cerebellar circuits provide a “universal cerebellar transform” that applies across motor and non-motor domains, then damage to the cerebellum should cause a similar pattern of deficits across motor, cognitive, and limbic behaviors, or a “universal cerebellar impairment” ^6^. It is possible that the computational processes that the cerebellum uses to coordinate movement with dynamic internal and external contexts are also used for its communication with non-motor brain areas. Based on these ideas, it is predicted that uncoordinated behavior in both motor and non-motor domains could emerge after cerebellar damage.

From an inter-regional connectivity perspective, it is intriguing to consider what the role of granule cells is in transforming mossy fiber information from cognitive brain areas, how this information is integrated with other inputs to granule cells, and how the information is fed forward to the rest of the brain. Future experimental work hopefully will reveal how the lessons we are learning about granule cells apply to long- and short-range connections and thereby shed further light on the fundamental contributions of granule cells to brain function and behavior.

## Summary

In the last few years, we have learned a great deal about how granule cells develop, form connections, and function. Perhaps the main lesson is that granule cells are surprisingly patterned in many aspects of their biology, from their lineage, gene expression, and proliferation through to their connectivity, function, and contributions to behavior. Multiple levels of clustering could be synonymous with flexibility—the sheer number of granule cells could demand multiple degrees of freedom with which to shape the developing cerebellum and control its circuits during motor and non-motor behaviors. Paradoxically, though, patterning also seems to serve as a platform for building cohesiveness into the network such that large populations can respond to the same stimuli. Based on their strong impact on development, function, and behavior, it is safe to say that there is power in numbers for this little cell type!

## Abbreviations

Atoh1, mouse homologue of atonal 1; Bmp, bone morphogenetic protein; Cux2, cut-like homeobox 2; Gli2, glioma-associated oncogene family zinc finger 2; Lmx1a, LIM homeobox transcription factor 1 alpha; Meis1, Meis homeobox 1; Pax6, paired box 6; Ptf1a, pancreas transcription factor 1 a; Shh, sonic hedgehog; Tbr2, T-box brain 2 (eomes/eomesodermin); Wls, wntless (Wnt ligand secretion mediator)
